# RGD-conjugated rod-like viral nanoparticles on 2D scaffold improve bone differentiation of mesenchymal stem cells

**DOI:** 10.3389/fchem.2014.00031

**Published:** 2014-05-27

**Authors:** Pongkwan Sitasuwan, L. Andrew Lee, Kai Li, Huong Giang Nguyen, Qian Wang

**Affiliations:** ^1^Department of Chemistry and Biochemistry, University of South CarolinaColumbia, SC, USA; ^2^Integrated Micro-Chromatography SystemsColumbia, SC, USA; ^3^Weifang Entry-Exit Inspection and Quanrantine BureauWeifang, Shandong, China; ^4^Department of Chemistry, The Institute of Catalysis for Energy Processes, Northwestern UniversityEvanston, IL, USA

**Keywords:** viral nanoparticles, RGD peptide, click chemistry, osteogenesis, bone mesenchymal stem cells

## Abstract

Viral nanoparticles have uniform and well-defined nano-structures and can be produced in large quantities. Several plant viral nanoparticles have been tested in biomedical applications due to the lack of mammalian cell infectivity. We are particularly interested in using *Tobacco mosaic virus* (TMV), which has been demonstrated to enhance bone tissue regeneration, as a tunable nanoscale building block for biomaterials development. Unmodified TMV particles have been shown to accelerate osteogenic differentiation of adult stem cells by synergistically upregulating bone morphogenetic protein 2 (BMP2) and integrin-binding bone sialoprotein (IBSP) expression with dexamethasone. However, their lack of affinity to mammalian cell surface resulted in low initial cell adhesion. In this study, to increase cell binding capacity of TMV based material the chemical functionalization of TMV with arginine-glycine-aspartic acid (RGD) peptide was explored. An azide-derivatized RGD peptide was “clicked” to tyrosine residues on TMV outer surface via an efficient copper(I) catalyzed azide-alkyne cycloaddition (CuAAC) reaction. The ligand spacing is calculated to be 2–4 nm, which could offer a polyvalent ligand clustering effect for enhanced cell receptor signaling, further promoting the proliferation and osteogenic differentiation of bone marrow-derived mesenchymal stem cells (BMSCs).

## Introduction

**Figure 1:**
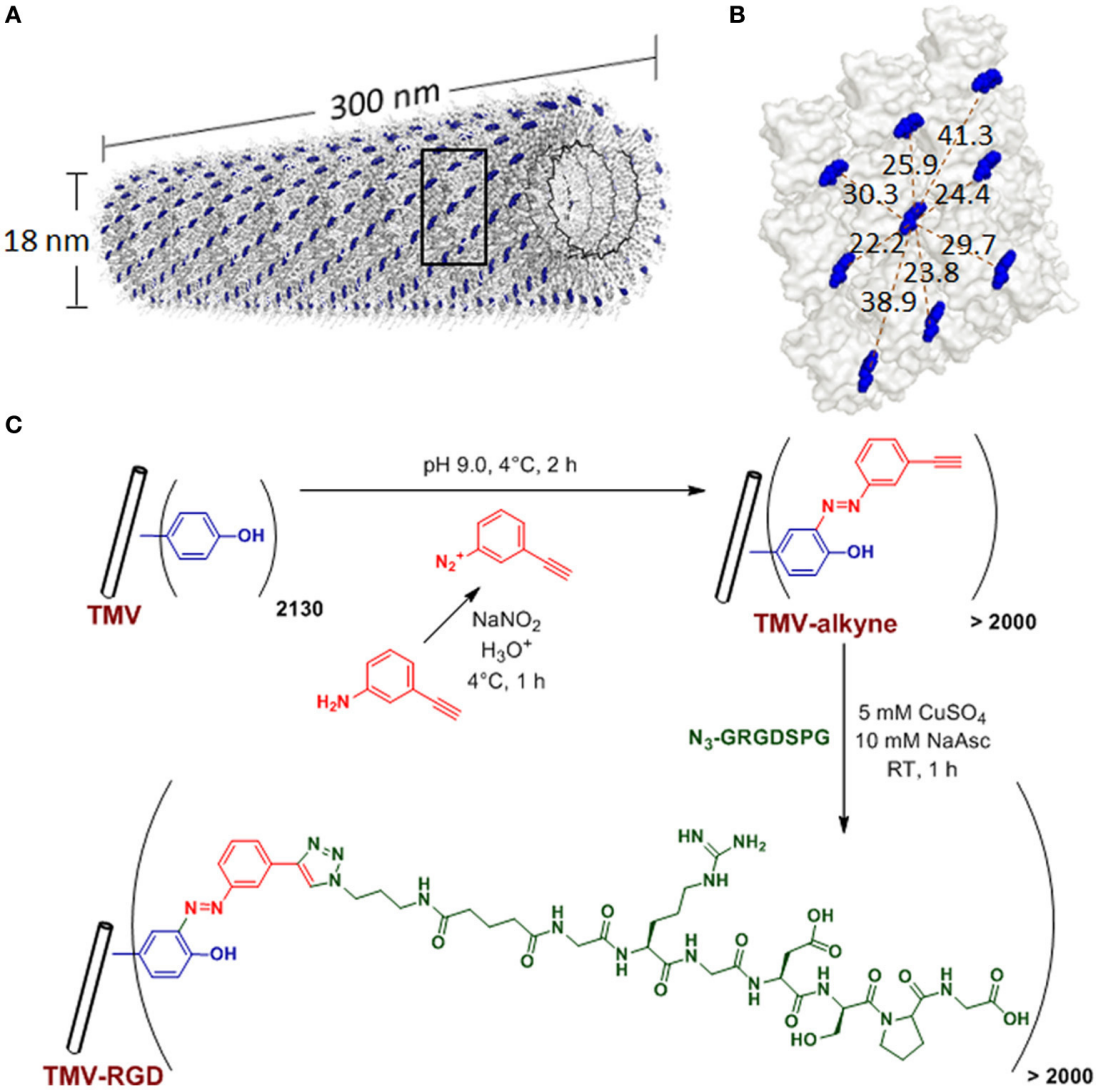
**TMV structure and bioconjugation scheme. (A)** Computer reconstructed image showing TMV structure using PyMOL with coordinates from Protein Data Bank. The single stranded RNA inside TMV particle is represented by the black helix. The tyrosine 139 (Y139) residues of individual TMV coat proteins subunits are colored in blue while all other amino acid residues are washed out in gray. **(B)** An enlarged portion of TMV coat protein [from the boxed area in **(A)**], showing possible distances (dashed red lines, measured in Angstroms) among the blue Y139. **(C)** Scheme of the TMV bioconjugation reaction to tether RGD peptides via CuAAC reactions to alkyne-functionalized Y139 residues.

Plant viral nanoparticles are meta-stable, readily available, monodisperse, and structurally uniform bionanoparticles. Such plant-derived viral particles have gained great interest in nano- and biomedical applications. *Tobacco mosaic virus* (TMV) is among the most commonly used plant viruses, having a rod-shape measuring 300 nm in length and 18 nm in diameter (Figure 1A). The viral capsid consists of 2130 identical coat protein subunits assembled in a helical structure around the single stranded genomic RNA. The production of TMV is cost effective and the resulting viral particles are highly uniform in size. TMV nanoparticles have been demonstrated as powerful building blocks that can be efficiently functionalized via both genetic (Jiang et al., 2006; McCormick et al., 2006; Lee et al., 2012b) and chemical (Schlick et al., 2005; Bruckman et al., 2008) modifications. Due to its identical subunits and regular structure, the same modification occurs on each individual subunit to yield a polyvalent and monodisperse display of ligands within a single TMV particle.

The tripeptide arginine-glycine-aspartic acid (RGD) present in many adhesive proteins in the extracellular matrix (ECM) is a well-known general cell recognition motif via the cell surface integrin receptors (Ruoslahti and Pierschbacher, 1987). These proteins include fibronectin, vitronectin, osteopontin, collagen, thrombospondin, fibrinogen, and von Willebrand factor (Ruoslahti and Pierschbacher, 1987). Generally, the incorporation of RGD sequence into artificial scaffolds increases initial cell adhesion to the scaffold and cell spreading, thus improving tissue regeneration process. It has been suggested that the spacing between RGD motifs needs to be less than 440 nm to mediate fibroblast adhesion and spreading and less than 140 nm to mediate focal adhesion assembly (Massia and Hubbell, 1991). Later, it was discovered that the RGD cluster spacings have a threshold less than 60 nm in order for NR6 fibroblasts to form focal adhesion and stress fiber (Maheshwari et al., 2000). Moreover, the threshold spacings are lower (closer) if there is less RGD clustering. In other words, at the same surface RGD density, the surface with RGD clusters present would provide greater cell adhesion strength (Maheshwari et al., 2000). Another study illustrated that integrin-mediated signaling requires RGD spacing of less than 58 nm (Cavalcanti-Adam et al., 2006). A more recent study showed that focal adhesion complexes between cell-membrane integrins and cytoskeleton, responsible for signal transduction from external stimuli to the cell, were formed when RGD spacing is less than 44 nm in endothelial cells (Le Saux et al., 2011). Specifically in the case of osteogenesis, there are several studies illustrating that the incorporation of RGD sequence into biomaterials improved bone differentiation and regeneration (Shin et al., 2004, 2005; Anderson et al., 2010; Peng et al., 2011; Qu et al., 2011). Especially, it was recently emphasized that a local clustering of RGD ligands is more essential than global RGD density (Deeg et al., 2011; Wang et al., 2013b). This clustering effect is believed to occur at an integrin-binding spacing less than an integrin itself, which has a size of 8–12 nm.

Many viral nanoparticles have been employed to achieve RGD-displaying clustering, such as M13 bacteriophage (Souza et al., 2006; Rong et al., 2008; Merzlyak et al., 2009; Chung et al., 2010; Wang et al., 2013a), *Cowpea mosaic virus* (Hovlid et al., 2012), *Turnip yellow mosaic virus* (Zeng et al., 2011; Zan et al., 2012), including genetically modified TMV with RGD peptides (Luckanagul et al., 2012; Lee et al., 2012a,b). However, to guarantee a successful assembly of final mutant TMV particles, TMV can only tolerate limited sequence diversity and length of the genetic peptide insertion (Lee et al., 2012b). Therefore, we explore the feasibility of TMV functionalization with the copper catalyzed alkyne-azide cycloaddition (CuAAC) reaction to display RGD peptides. In addition, the influence of RGD-presenting TMV, where RGD clustering is present (RGD spacing of 2–4 nm) (Figure 1B), on the osteogenic potential of bone marrow derived mesenchymal stem cells (BMSCs) is investigated.

## Materials and methods

### TMV isolation, bioconjugation, and characterization

TMV was isolated and purified according to a protocol previously reported (Kaur et al., 2010a,b). The schematic representation of TMV bioconjugation is shown in Figure 1C. The RGD-azide peptide was synthesized using solid-phase peptide synthesis. The peptides were purified with FPLC and characterized by LC/ESI mass spectrometry. The CuAAC reaction to modify tyrosine residues on the exterior surface of TMV is performed according to protocols established by Schlick et al. (2005) and Bruckman et al. (2008) with slight modifications. Briefly, diazonium salt was synthesized by mixing 16 parts of 0.3 M *p*-toluenesulfonic acid, three parts of 0.67 M 3-aminophenylacetylene, and one part of 3 M sodium nitrite as published previously (Schlick et al., 2005). TMV was treated with 25 molar excess of the diazonium salt generated *in situ* from 3-aminophehylacetylene at 4°C in a pH 9.0 buffer solution to form alkyne grafted TMV particle. CuAAC reaction was used to conjugate RGD-azide peptide to TMV particle (Wang et al., 2003). The CuAAC reaction was done with concentration of TMV-alkyne at 3 mg/mL and peptide-azide at 4 mg/mL in Tris HCl buffer (10 mM, pH 7.8), in the presence of 5 mM copper (II) sulfate and 10 mM sodium acetate. After 1 h incubation at room temperature, TMV-RGD was purified via a 10–50% sucrose gradient from which the light scattering region was collected. The modified virus was then pelleted using ultracentrifugation at 160,000 g for 2.5 h at 4°C. The pellet was dissolved in potassium phosphate buffer (10 mM, pH 7.4). MALDI-TOF mass spectrometry was used to confirm the modifications. The integrity of modified TMV particles was confirmed by AFM and TEM. The virus solutions were dialyzed against water prior to substrate coating.

### Preparation of virus coated substrates

For cell culture experiments, 3-aminopropyltriethoxysilane (APTES) coated slides (Lab Scientific Inc.) were cut into 1.5 cm^2^ wafers. The wafers were washed with ethanol before use. For virus coating, each wafer was coated with 0.2 mL of 0.2 mg/mL TMV or TMV-RGD solution diluted in water and the coated substrates were dried overnight in a sterile biosafety cabinet. The virus coverage on the wafers was characterized using tapping-mode AFM images using a NanoScope IIIA MultiMode AFM (Veeco). Si tips with a resonance frequency of approximately 300 kHz, a spring constant of about 40 N m^−1^ and a scan rate of 1.0 Hz were used.

### BMSC isolation and expansion

Primary BMSCs were isolated from the bone marrow of young adult 80 g male Wister rats (Harlan Sprague Dawley, Inc.). The procedures were performed in accordance with the guidelines for animal experimentation by the Institutional Animal Care and Use Committee, School of Medicine, University of South Carolina. Cells were maintained in growth medium [DMEM supplemented with 10% fetal bovine serum (FBS), penicillin (100 U/mL), streptomycin (100 μg/mL), and amphotericin B (250 ng/mL)] and passaged no more than four times after isolation. To induce osteogenesis, growth media was replaced with osteogenic media consisting of DMEM supplemented with 10% FBS, penicillin (100 U/mL), streptomycin (100 μg/mL), amphotericin B (250 ng/mL), 10 mM sodium β-glycerophosphate, L-ascorbic acid 2-phosphate (50 μg/mL), and 10^−8^ M dexamethasone. Media was replenished every 3–4 days.

### Cell proliferation

Substrates coated with TMV and TMV-RGD were seeded with 4 × 10^4^ cells per substrate and cells were allowed to attach overnight in growth media. The media was then replaced with osteogenic media and cultured for 22 days. CellTiter Blue® assay (Promega) was used to determine number of cells at 0, 2, 4, 9, 12, 16, and 22 days after osteogenic induction. Cell proliferation was determined by normalizing CellTiter Blue fluorescence intensities against initial signal intensity on day 0, which is the day of osteogenic induction.

### Quantitative real-time RT-PCR analysis (RT-qPCR)

Virus coated wafers were seeded with 4 × 10^4^ cells per wafer and cells were allowed to attach overnight in growth media. The unseeded cells were used as a control to normalize the change in gene expression. The media was replaced with osteogenic media and cultured for 7, 14, and 21 days. The cell cultures were terminated at these time points and total RNA was extracted using RNeasy mini purification kit (Qiagen). The quality and quantity of the extracted RNA was analyzed using Bio-Rad Experion (Bio-Rad Laboratories) and was reverse transcribed by using qScript™ cDNA Supermix (Quanta Biosciences). RT-qPCR (iQ5 real-time PCR detection system Bio-Rad Laboratories) was done by the method described as: 60 cycles of PCR (95°C for 20 s, 58°C for 15 s, and 72°C for 15 s), after initial denaturation step of 5 min at 95°C, by using 12.5 μL of iQ5 SYBR Green I Supermix, 2 pmol/μL of each forward and reverse primers and 0.5 μL cDNA templates in a final reaction volume of 25 μL. Glyceraldehyde 3-phosphate dehydrogenase (GAPDH) was used as the housekeeping gene. Data collection was enabled at 72°C in each cycle and C_T_ (threshold cycle) values were calculated using the iQ5 optical system software version 2.1. The expression levels of differentiated genes and undifferentiated genes were calculated using Pfaffl's method (M. W. Pfaffl, G. W. Horgan, and L. Dempfle, Relative expression software tool) for group-wise comparison and statistical analysis of relative expression results in real-time PCR, using GAPDH as the reference gene. Quantification of gene expression was based on the C_T_ value for each sample which was calculated as the average of three replicate measurements for each sample analyzed. “Pair Wise Fixed Reallocation Randomization Test” was performed on each sample and a value of *p* < 0.05 was regarded as significant. The primers used for RT-qPCR are shown in Figure S1.

### Alkaline phosphatase activity

After 14 days in osteogenic cultures, CellTiter Blue® assay (Promega) was used to determine number of cells in each sample 1 h prior to cell fixation. BMSCs seeded on TMV and TMV-RGD were fixed with 4% paraformaldehyde for 15 min at room temperature. To determine alkaline phosphatase (ALP) activity, each fixed samples were incubated in 500 μL of 1-Step *p*-nitrophenyl phosphate solution (Thermo Scientific) for 15 min at room temperature. Then the solution was transferred to a new microfuge tube with 250 μL of 2 N NaOH and the absorbance at 405 nm was measured. The measured ALP activity from each sample was normalized to the corresponding cell number.

### Alizarin red staining

To visualize calcium deposition and to confirm osteogenic differentiation, fixed samples at day 14 were stained with 0.1% Alizarin red solution (Sigma-Aldrich) pH 4.1–4.5 for 30 min in the dark. The samples were washed with water (18.2 MΩ) prior to imaging.

## Results

**Figure 2:**
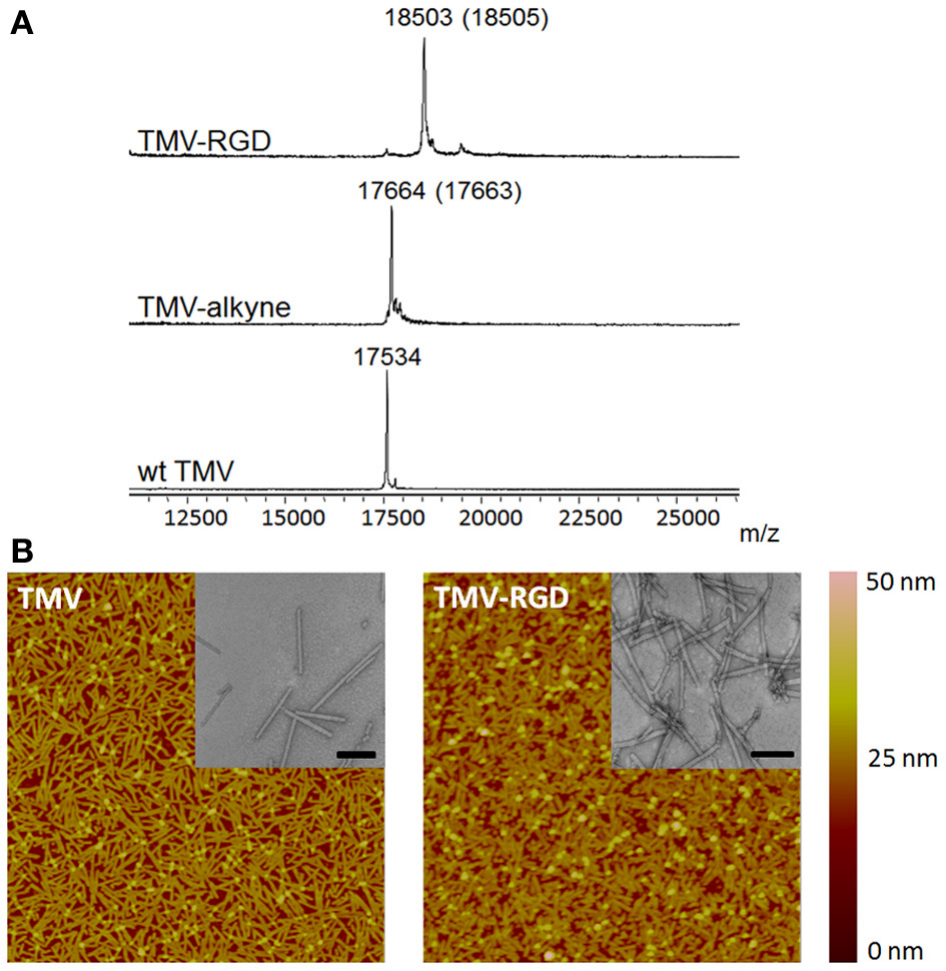
**Characterization of TMV particles. (A)** MALDI-TOF MS spectra of the subunit protein of wild type TMV (17534 m/z), TMV-alkyne (17664 m/z), and the CuAAC reaction product TMV-RGD (18503 m/z). The numbers in parentheses refer to the theoretical masses. **(B)** The morphology of TMV particles before (TMV) and after (TMV-RGD) bioconjugation visualized by AFM and TEM (insets). Scan areas of AFM are 5 × 5 μm; and scale bars of TEM are 200 nm.

The tyrosine residues (Y139) of TMV are viable for chemical ligation using electrophilic substitution reaction at the *ortho*-position of the phenol ring with diazonium salts (Schlick et al., 2005). TMV subunits are assembled in a highly regular helical structure, which resulted in uniform spacing between two subunits down to a nanometer scale. From coordinates provided by Protein Data Bank, the distance between neighboring Y139 residues of TMV coat proteins is calculated to be 2–4 nm apart as shown in Figure 1B. The CuAAC reaction has been confirmed to be a very efficient way to display functional groups on TMV in a controllable manner (Bruckman et al., 2008). Following the reported protocol (Schlick et al., 2005; Bruckman et al., 2008). We first prepared the alkyne derived TMV particles (Figure 1C). As shown in the MALDI-TOF MS analysis (Figure 2A), the peak of the coat protein (m/z 17534) disappeared after the diazonium coupling resulting in the TMV-alkyne product (m/z 17664), consistent with a previous report (Lee et al., 2012b). Similarly, the MALDI-TOF MS analysis indicated the correct mass shift for TMV-RGD confirmed the completion of the sequential CuAAC reaction (Figure 2A). The integrity of TMV-RGD was confirmed by AFM and TEM (Figure 2B). AFM images illustrated that the majority of TMV-RGD remained rod-shaped particles after the bioconjugation. The diameter observed under TEM ranged from 15–20 nm with lengths measuring approximately 300 nm, indicating the particles are intact after the two-step CuAAC reaction to Y139 (Figure 2B).

The effect of RGD-displaying TMV, as a polyvalent scaffold, on bone differentiation was explored. It has been demonstrated that unmodified TMV substrate accelerated osteogenesis by 7 days compared to standard tissue culture polystyrene (TCPS) control (Kaur et al., 2010a). The chemical incorporation of phosphate functional groups to TMV further enhanced bone differentiation of BMSCs (Kaur et al., 2010b). However, the interaction between BMSCs and TMV substrates were weak since the focal adhesion complexes were found to be significantly smaller than the control (Sitasuwan et al., 2012). Since RGD ligand is known to promote cell attachment, the incorporation of RGD motifs into TMV based substrate is expected to increase the initial cell adhesion.

**Figure 3:**
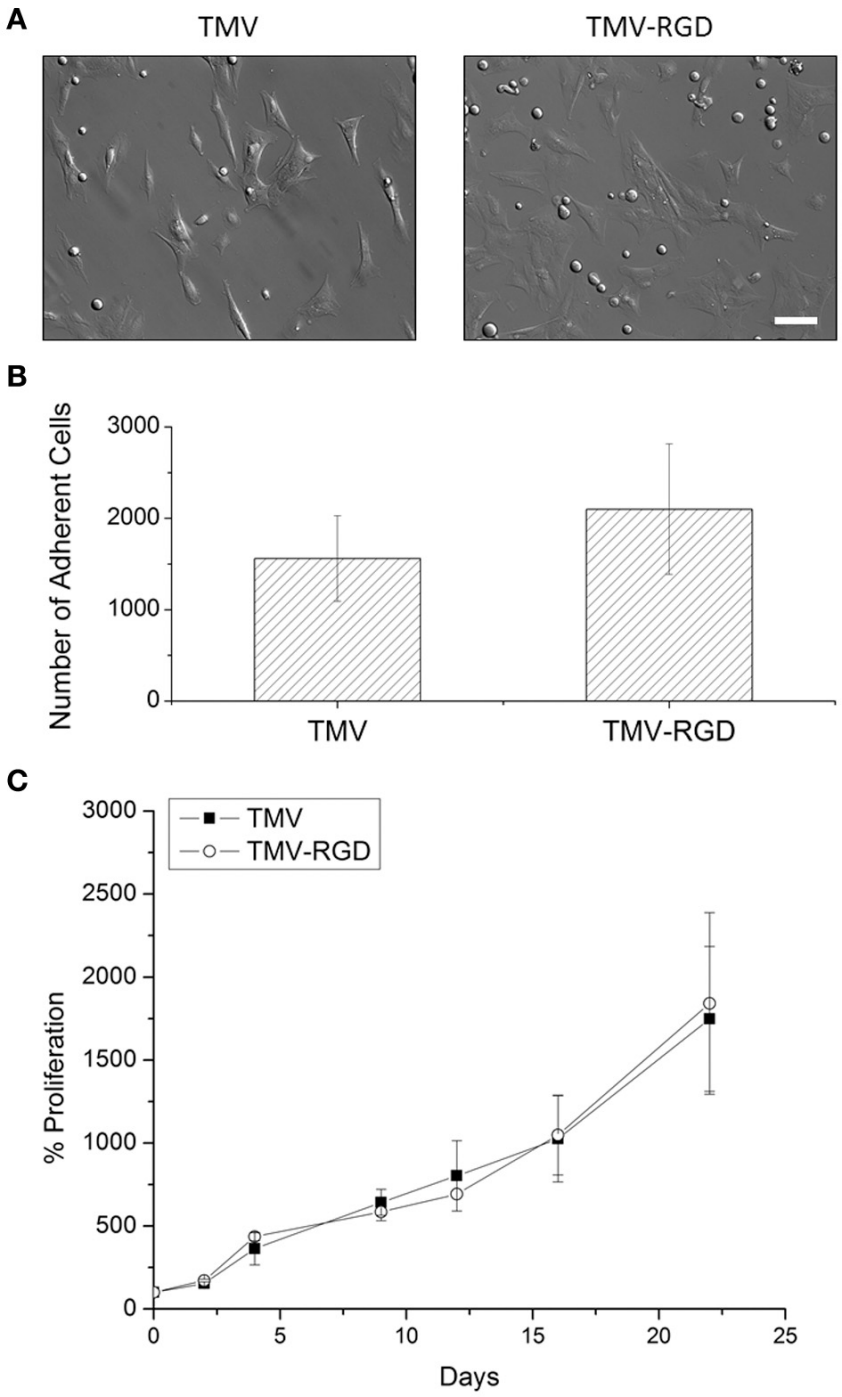
**BMSC adhesion and viability on virus scaffolds. (A)** Optical images of BMSC attached on virus substrates after 24-h seeding in primary media. Scale bar is 100 μm. **(B)** Number of adherent BMSCs on different virus substrates after 24-h seeding in primary media. **(C)** Proliferation percentage of BMSCs on TMV and TMV-RGD substrates over 22 days in osteogenic media. Error bars indicate ±1 SD.

Prior to cell culture experiments, TMV and TMV-RGD were dialyzed several times against ultrapure water, due to concerns that residual Cu^I^ from the CuAAC reaction will affect cell viability. Optical images after 24 h of cell seeding in Figure 3A revealed that BMSCs can adhere and spread on both TMV and TMV-RGD substrates. There were slightly more cells visualized on TMV-RGD compared to TMV coated surface. The numbers of adherent cells were calculated to elucidate the result in a quantitative manner (Figure 3B). As expected, the average cell numbers on TMV-RGD was higher than that of TMV. To verify that TMV-RGD does not have cytotoxicity from residual Cu^I^, the proliferation of BMSCs on both virus scaffolds was examined over 22 days in osteogenic conditions (Figure 3C). The proliferation percentage of each sample was calculated based on the initial cell attachment as a 100%. BMSCs proliferated comparably well on both virus substrates, thus TMV-RGD did not exhibit any cytotoxicity to the *in vitro* cell culture.

**Figure 4:**
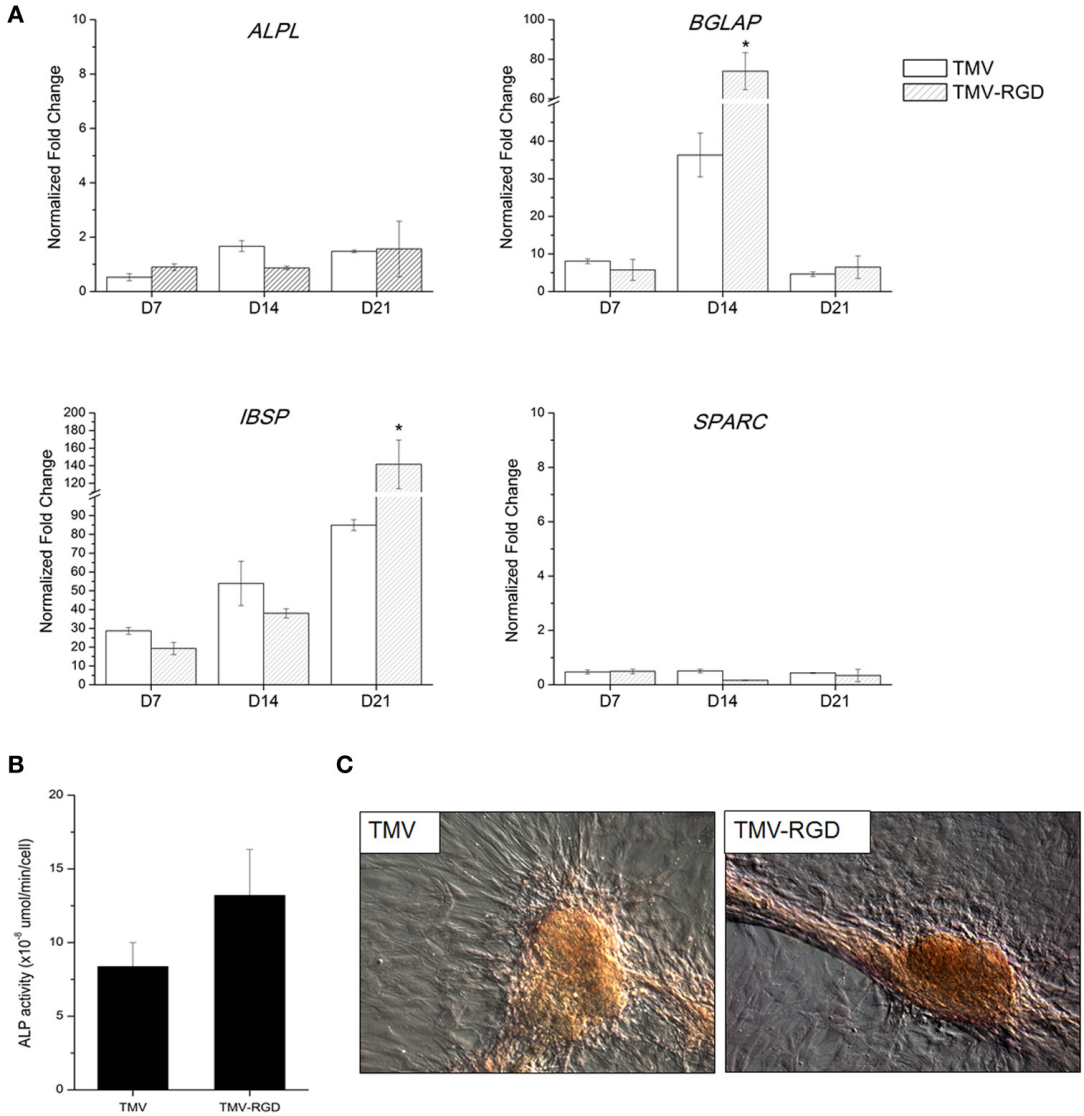
**Osteogenic differentiation of BMSCs on TMV and TMV-RGD substrates. (A)** Osteo-specific gene expression profiles of BMSCs on TMV and TMV-RGD under osteogenic conditions over 21 days. For each sample, the profiles show 3 time points at 7, 14, and 21 days. BMSCs on TMV-RGD substrates have significantly higher *BGLAP* at day 14 and *IBSP* at day 21 when compared with unmodified TMV substrates. Error bars indicate ±1 SD. ^*^p < 0.05. **(B)** Alkaline phosphatase activity of cells on TMV and TMV-RGD at day 14. BMSCs on TMV-RGD substrates have a slight increase in enzyme activity compared to cells on TMV. However, the difference is not statistically significant. **(C)** Optical images of Alizarin red S staining of cells cultured for 14 days on TMV or TMV-RGD substrate showing calcium deposition in red colour.

The differentiation potential of BMSCs was studied in order to substantiate the effect of RGD incorporation on osteogenesis. First, osteo-specific gene expression levels were quantified by RT-qPCR (Figure 4A). The expression levels are presented as fold change compared to BMSCs at day 0. There was no significant difference in the gene expression levels of alkaline phosphatase (*ALPL*), osteonectin (*SPARC*), and osteopontin (*SPP1*) over the 3 weeks. Another osteo-specific gene examined is osteocalcin (*BGLAP*), the most common marker of mature osteoblast, as this protein is only synthesized by fully differentiated osteoblasts (Fujisawa and Tamura, 2012). BGLAP is rich in acidic amino acids that are responsible for its high affinity to calcium ions (Fujisawa and Tamura, 2012), which are eventually accumulated in mineralized bone matrix by binding specifically to hydroxyapatite crystals (Owen et al., 1990). It has been documented that *BGLAP*, which is normally peaked at day 21 on standard tissue culture plate substrate, is peaked at day 14 when BMSCs are grown on TMV scaffold suggesting that TMV substrates accelerate the bone differentiation process by 7 days (Kaur et al., 2010a). The gene expression analysis of BMSCs on unmodified TMV scaffolds in Figure 4A was in agreement with the previous report (Kaur et al., 2010a), where *BGLAP* gene expression level was peaked at day 14 indicating a complete mineralization of mature osteoblasts. As a comparison, the incorporation of RGD peptide into TMV subunits also significantly increased *BGLAP* gene expression level at day 14 (Figure 4A). The gene expression level of integrin-binding bone sialoprotein (*IBSP*), a secreted ECM protein required for hydroxyapatite formation as well as collagen binding in mineralized tissues (Ogata, 2008), was also evaluated. IBSP is synthesized just before calcification (Fujisawa and Tamura, 2012) and real time PCR results from the previous study indicated that BMSCs on TMV substrates had significantly higher *IBSP* mRNA expression within 24 h while there was no change in *IBSP* mRNA expression levels in BMSCs on TCPS control (Sitasuwan et al., 2012). In this experiment, *IBSP* expression levels in cells on both TMV and TMV-RGD were highly upregulated during the culture period (Figure 4A). However, BMSCs grown on TMV-RGD scaffolds expressed remarkably higher level of *IBSP* mRNA at day 21 when compared to those on native TMV scaffolds at the same time (Figure 4A).

In addition to the analysis of osteo-specific gene expressions, ALP activity was assessed. ALP is an early marker of osteogenesis and its activity mediates matrix mineralization. Although there was no difference in *ALPL* mRNA expressions at day 14 for cells grown on TMV compared to those on TMV-RGD as shown in Figure 4A, ALP enzyme activity assay showed a slight increase in BMSCs grown on TMV-RGD (Figure 4B). The staining for calcium deposition was also performed at day 14. A stronger staining was observed on TMV-RGD samples suggesting higher mineralization level (Figure 4C). This observation could be supported by previously mentioned increases in both *BGLAP* and *IBSP* mRNA expression levels (Figure 4A), possibly facilitating the formation of hydroxyapatite crystals leading to mineralized matrix.

## Discussion

TMV has previously been shown to be an effective scaffold that accelerates bone differentiation of stem cells when coated on a 2D substrate and provides support for cell differentiation in 3D alginate hydrogels (Kaur et al., 2010a,b; Luckanagul et al., 2012; Sitasuwan et al., 2012). While the underlying mechanism is still unclear, the level of the potent osteogenic induction protein, bone morphogenetic protein 2 (BMP2), was significantly increased within 24 h for cells cultured on substrates with TMV coating compared to cells cultured on uncoated substrates or cells supplemented with TMV in suspension (Sitasuwan et al., 2012). One possible interpretation of this observation is that the topographical features created by TMV coating, compared to TMV in solution, plays a major role in the accelerated osteogenic differentiation.

Cells are capable of sensing the surrounding microenvironment, which provides both biochemical and biophysical cues, leading to downstream signaling cascades responsible for diverse cellular processes, such as adhesion, migration, proliferation, and apoptosis (Curtis and Wilkinson, 1997). For example, the nanoscale roughness of titanium surface implant can positively affect implant integration and bone differentiation (Lossdörfer et al., 2004; Mozumder et al., 2012; Olivares-Navarrete et al., 2012; Zhuang et al., 2012). In addition, the incorporation of growth factors (Crouzier et al., 2011; Wang et al., 2011; Kopf et al., 2012; Lee et al., 2013), adhesion ligands (Shin et al., 2005; Duggal et al., 2009; Qu et al., 2011), and osteoinductive compounds (Shu et al., 2003; Verma et al., 2010; Hao et al., 2011; Cameron et al., 2013) into biomaterial surface can further accelerate the bone formation process. Given the lack of affinity of native TMV viral particles to mammalian cell surface, which resulted in low initial cell adhesion (Sitasuwan et al., 2012), we hypothesized that combining the topological features offered by TMV with cellular adhesion molecules could synergistically enhance the bone formation process of stem cells.

In our previous study, we reported the genetic incorporation of RGD peptides on the coat protein of TMV particles and the resulting mutant virus could enhance the adhesion of BMSCs (Lee et al., 2012b) and accelerate the stem cell differentiation in serum free, chemically defined osteogenic media (Lee et al., 2012a). However, the genetic insertion suffers from the limited length and sequence diversity of the fusion peptides. To guarantee a successful assembly of final mutant TMV particles, TMV can only tolerate limited sequence diversity and length of the genetic peptide insertion. In this work, we demonstrate the feasibility of modulating mesenchymal stem cell differentiation on TMV-based scaffolds modified by CuAAC reaction. TMV was successfully modified with more than 95% conversion and the integrity of the virus particles was preserved. Scaffolds coated with TMV-RGD slightly improved initial BMSC adhesion, while maintaining the same cell proliferation rate with those coated with native TMV. The osteogenic differentiation of BMSCs was enhanced on TMV-RGD substrates since an increase in *BGLAP* and *IBSP* gene expression levels as well as mineralization level was observed, similar as TMV-RGD genetic mutant. However, the chemically tailored TMV particles have shown greater stability than TMV-RGD mutant particles.

It was expected that the displayed RGD peptides on TMV particles could lead to significantly greater cell attachment. However, it only facilitated a slight increase in the initial BMSC adhesion. A possible explanation is that TMV-RGD tends to aggregate leading to hindered ligand display. A precipitate was observed after the CuAAC reaction to functionalize TMV-alkyne with RGD peptide. Similarly, the rapid decomposition and aggregation of an icosahedral plant virus was previously observed when the virus is decorated with triazole with the presence of free copper ions in solution (Wang et al., 2003). Future studies will focus on controlling ligand density and using a variety of ligands including small molecules and other peptides on viral scaffold to study structure-property relationship and modulate cell behavior. It is also important that a recent development of copper-free Click reaction (Lallana et al., 2011) may provide an alternative approach to chemically modify TMV without a concern about Cu^I^ contamination.

### Conflict of interest statement

The authors declare that the research was conducted in the absence of any commercial or financial relationships that could be construed as a potential conflict of interest.

## Supplementary material

Figure S1 **Primers used for RT-qPCR to measure gene expression levels**. ALPL, alkaline phosphatase; BGLAP, osteocalcin; IBSP, integrin-binding sialoprotein; SPARC, osteonectin; SPP1, osteopontin.
