# Supplementary material for: RGD-conjugated rod-like viral nanoparticles on 2D scaffold improve bone differentiation of mesenchymal stem cells
Source: Front Chem. 2014 May 27;2:31. doi: 10.3389/fchem.2014.00031 (PMC4034042; doi:10.3389/fchem.2014.00031)
Supplement: Figure S1 — Primers used for RT-qPCR to measure gene expression levels. ALPL, alkaline phosphatase; BGLAP, osteocalcin; IBSP, integrin-binding sialoprotein; SPARC, osteonectin; SPP1, osteopontin. [file Presentation1.PDF]

Gene	Sequence (5'-3')
ALPL	Forward: ACCTCGAGCAGGAACAGAAGTTTGCT
	Reverse: TTCCATGATGGTTGCAGGGTCTGGA
BGLAP	Forward: AAAGCCCAGCGACTCT
	Reverse: CTAAACGGTGGTGCCATAGAT
GAPDH	Forward: ACTAAAGGGCATCCTGGGCTACACTGA
	Reverse: TGGGTGGTCCAGGGTTTCTTACTCCTT
IBSP	Forward: TCTGAACGGGTTTCAGCAGACGA
	Reverse: GCCATGCCCCTTGTAGTAGCTGTA
SPARC	Forward: AAACTTGGGCAAGGACGCTGTGA
	Reverse: ATGCCTCCCCTTGGCTCACCAAAAT
SPP1	Forward: GACGGCCGAGGTGATAGCTT
	Reverse: CATGGCTGGTCTTCCCGTTGC
